# Smart Environmental Monitoring and Assessment Technologies (SEMAT)—A New Paradigm for Low-Cost, Remote Aquatic Environmental Monitoring

**DOI:** 10.3390/s18072248

**Published:** 2018-07-12

**Authors:** Jarrod Trevathan, Ron Johnstone

**Affiliations:** 1 School of Information and Communication Technology, Griffith University, Meadowbrook Logan 4131, Australia; 2 School of Earth and Environmental Science, University of Queensland, St Lucia Brisbane 4067, Australia; r.johnstone@uq.edu.au

**Keywords:** environmental monitoring, social enterprise, open source technologies, internet of things, wireless sensor networks, low-cost sensors

## Abstract

Expense and the logistical difficulties with deploying scientific monitoring equipment are the biggest limitations to undertaking large scale monitoring of aquatic environments. The Smart Environmental Monitoring and Assessment Technologies (SEMAT) project is aimed at addressing this problem by creating an open standard for low-cost, near real-time, remote aquatic environmental monitoring systems. This paper presents the latest refinement of the SEMAT system in-line with the evolution of existing technologies, inexpensive sensors and environmental monitoring expectations. We provide a systems analysis and design of the SEMAT remote monitoring units and the back-end data management system. The system’s value is augmented through a unique e-waste recycling and repurposing model which engages/educates the community in the production of the SEMAT units using social enterprise. SEMAT serves as an open standard for the community to innovate around to further the state of play with low-cost environmental monitoring. The latest SEMAT units have been trialled in a peri-urban lake setting and the results demonstrate the system’s capabilities to provide ongoing data in near real-time to validate an environmental model of the study site.

## 1. Introduction

With the continued urbanization of natural habitats, the management of precious waterways in and around cities is of paramount importance. In addition to the necessity for supplies of potable water in source catchments, there is also the need to ensure that water bodies associated with infrastructure and areas of habitation do not represent a pollutant or health risk to the associated communities [1]. 

The traditional periodic or episodic monitoring of these waters is increasingly becoming insufficient due to changes in licensing needs for industry and the need for improved predictive capacity for risk assessment and management. Concomitantly, environmental managers are progressively seeking to better understand the dynamic and complex interactions of hydrological processes and human influences, so that they are able to effectively act in a timely manner and meet community safety expectations. As noted in a number of reviews [2,3,4] traditional manual and many technical monitoring systems continue to be cost prohibitive and require human resources that are often limited. Accordingly, the spatial and temporal scope of the data collected is similarly limited. Subsequently, there continues to be a need for cheaper, cost effective monitoring systems capable of delivering real-time information suited to the response requirements of current and future management agencies. 

In 2009, the *Smart Environmental Monitoring and Assessment Technologies* (SEMAT) project [5,6,7,8] was conceived. SEMAT is an initiative to develop low-cost environmental monitoring systems for aquatic environments that can collect data remotely in near real-time. The core project aim was to dramatically reduce the cost of such systems using existing commercially available “*off the shelf*” technologies [9]. By doing this, the intent was to allow managers to increase both the temporal and spatial resolution of data collection and, therefore, meet the need to more accurately react to events and also better define the functional attributes of the environment being managed. This improved data collection would also allow for the development and more accurate validation of predictive models used to underpin decision-making. 

The initial SEMAT project clearly demonstrated that a low-cost approach to environmental monitoring is indeed possible and that it could deliver the levels of accuracy and system performance required by keystone agencies consulted at the time. The system was benchmarked through deployments at Moreton Bay and Heron Island in Queensland, Australia, to investigate hazardous algal blooms [2,7,8]. Since these deployments, the SEMAT technology has continued to evolve becoming smaller and more powerful (in line with Moore’s Law). Furthermore, the push towards programmable electronics has reduced the breadth of skillsets required to develop such environmental monitoring systems, increasing the access to this type of technology to a wider stakeholder community [9,10]. Furthermore, the increasing need to reduce environmental impacts from such technologies and to lessen electronic waste has driven systems development to consider the use of up-cycled and re-purposed components to reduce waste to landfill [11]. This has particular relevance for improving the uptake of such technologies in poorer countries where e-waste solutions and the need for cheap monitoring solutions co-exist. 

This paper discusses a new community engagement paradigm for the development of low-cost environmental monitoring systems and presents the latest developments for the SEMAT project from a physical and technological perspective. We review and refine the key goals for inexpensive aquatic environmental monitoring, analyse the core hardware and software systems and present a reproducible design for SEMAT environmental monitoring buoys. Furthermore, we discuss a new approach to production that directly aims to reduce the environmental footprint of the technology and also enhances the social and economic gains from its production and use. 

The deployment used to test and validate the latest SEMAT system was a lake subject to human and natural influences typical of the operational landscape for many end-users of the technology. The range of impacts included polluted water inputs from a nearby motorway, car park, golf course and residential properties, as well as disturbance by aquatic birds, changing water levels and intense weather events. The field test demonstrates that the latest SEMAT buoys achieve the stated goals and the system is ready to be deployed on a larger scale across wetlands, creeks, rivers and constructed water bodies such as water retention basins and aquaculture facilities. 

This paper is structured as follows: Section 2 outlines the related work, SEMAT’s revised/refocused design goals and describes a new community engagement paradigm for developing low-cost, remote aquatic monitoring systems. Section 3 proposes the SEMAT environmental monitoring open standard. Section 4 presents the results from a field validation deployment in a peri-urban lake; and Section 5 provides some concluding remarks and avenues for future work. 

## 2. Developing Low-Cost, Remote Aquatic Environmental Monitoring Systems

### 2.1. Problem Motivation and Related Work

Expense is the most limiting factor for undertaking aquatic environmental monitoring studies. Current approaches involve the use of expensive proprietary devices, or human involvement to either manually take water samples over a period of time, or to physically deploy logging devices which must be later retrieved. These are all costly and time consuming. Expensive proprietary devices greatly limit the number of devices that can be deployed and often also involve additional infrastructure to allow for communication and exchange. In the latter cases, a logger may have failed (destroying its data) without any indication of its condition until its retrieval (perhaps after several months of deployment) and manually collected data by sampling is time limited or snap shot; with some values not being known until well after the event. Apart from leading to fragmented data sets, these approaches provide limited opportunity to react to events as they are unfolding preventing proactive actions to limit the effects that might be realized in the field.

The ability to monitor an aquatic environment remotely and in *near real-time* (i.e., 5–15 min intervals) is an extremely useful and powerful tool [12]. Not only does this reduce the amount of time humans must spend out in the field (with the associated dangers and costs) but it also allows environmental models to be updated dynamically as new data is received. This can empower decision makers by informing them of the most appropriate actions to take depending on the phenomenon being studied.

**Table 1:** Research-Based Water Quality Monitoring Initiatives.

Research Initiative	Location
IMOS (*Integrated Marine Observing System*) [13]	Tasmania, Australia
GBROOS (*Great Barrier Reef Oceanic Observation System*) [14]	Great Barrier Reef, Queensland, Australia
LakeNet [15]	St. Mary’s Lake, USA
OceanSense [16]	Coastal waters of China
Klimat [17]	Baltic Sea, Sweden
SmartCoast [18]	River Lee, Cork, UK
ReCON (*Real-time Coastal Observation Network*) [19]	Lakes Michigan, Huron, Erie, USA
GLUCOS (*Great Lakes Urban Coastal Observing System*) [20]	Lake Michigan, USA

Over the last decade, this capability has become highly sought after and several major projects have been initiated using *Wireless Sensor Network* (WSN) [21,22] and *Internet of Things* (IoT) [23] technologies. Table 1 presents some examples of research initiatives for water quality monitoring. Such projects typically involved large government organizations, significant amounts of funding and substantial human resources to realize. Alternately, other projects were one-off initiatives by research institutions and the projects have since concluded many years ago with no further advancement on the state-of-the-art. While these initiatives were noble in their aspirations, expense again became the most significant limiting factor. Accordingly, the cost of an environmental monitoring unit prohibited how many of the units that could be deployed and therefore greatly compromised the spatial coverage that could be achieved in data collection. From an environmental modelling perspective and the need to identify change at multiple scales, the level of data acquisition that these initiatives were able to achieve was still quite limiting.

Several proprietary products have been made commercially available through scientific instrumentation companies. Table 2 provides some examples of these products. While highly precise, these approaches tend to be ridged and costly in the sense that the end user of the system can only use the equipment in the way determined by the vendor and the capital investment is high. Furthermore, the end user is reliant on the vendor for all maintenance, adaptations and modifications to the equipment.

Within this context SEMAT aimed to apply a new paradigm to low-cost aquatic environmental monitoring. The intent was to see whether off-the-shelf commercially available componentry could be assembled in standardized formats for the purpose of such monitoring. A series of prototype SEMAT buoys were constructed using this approach and two whole-of-system deployments were undertaken in Moreton Bay and Heron Island lagoon in Queensland Australia [2,7,8,29]. These deployments demonstrated that it was not only feasible to construct suitable environmental monitoring systems using a commodity-based approach but also to obtain the enhanced temporal and spatial resolution which greatly increased the quality and value of the data. In addition, the SEMAT approach reasons that the system is able to provide readings from several lower-cost units to inform the scientist as to the best locations and times to commit the more expensive, highly precise manual investigations should they be required.

Concurrent work by Albaladejo et al. 2012 [30] also pursued a low-cost sensor buoy system. They designed a device for monitoring near-shore, shallow marine environments as part of the Spanish National research project “Observatorio Oceanografico Costero de la Region de Mucia.” Their buoy recorded temperature, water pressure and atmospheric pressure remotely using a light weight but marine-robust platform. The system was the first to use 3.7 volt LiPo batteries with small form factor solar panels. They deployed the system over several months around the Mar Menor Lagoon in the Mediterranean Sea with relatively successful results. However, this initiative appears to have concluded with no further studies having been undertaken since 2011.

Since 2012, many other initiatives have arisen that attempt to take a different approach to the traditional methods of environmental monitoring. The Cave Pearl Project [10] focuses on using Arduino microcontrollers to construct extremely low power consumption logging devices. The loggers use Arduino-compatible sensors and custom-made logger housings in order to keep the cost down. At the time of writing, the project claims that a logger can run for over a year with three AA batteries for approximately $100 USD. These loggers do not provide telemetry and therefore must be retrieved from the site in order to download the data. However, the project blog contains useful construction guides for equipment and much anecdotal evidence about the realities of undertaking deployments.

Lockridge et al. [31] present a low-cost, drifting sonde that remotely transmits various environmental and geolocation parameters from its attached sensors and GPS module. The sonde took measurements on temperature and conductivity over several weeks while adrift in the ocean. However, the system did not use solar power. Similar to the Cave Pearl Project, this proposal is based on the widely available Arduino microcontroller platform. Although low-power micro controller units exist, Arduino’s simplicity and open source nature makes it attractive for getting environmental monitoring applications running easily.

In this vein, Sadler et al. [9] also proposed an architecture for an environmental monitoring system based on Arduino. They took the SEMAT-approach for selecting off-the-shelf components to achieve lower costs. They interfaced the Arduino with a DHT22 Humidity/Temperature sensor and SDI-12 pressure transducer. The data was sent via GPRS modem to a server. The system was powered by a 4000 mAh lithium ion battery and 6 W solar panel. Sadler et al. also called for an open-source, open-data approach to development. However, a physical buoy was not constructed for their system and no actual field validation tests were conducted in water.

Although there are still many technical challenges to overcome, it is apparent that the technology now exists to make remote environmental monitoring systems dramatically less expensive and the low-cost approach is gaining traction. 

### 2.2. SEMAT’s Design Goals (Revisited)

Trevathan et al. [8] outlined the initial goals for SEMAT, evolution of the SEMAT buoys and trial deployments. Some associated publications [29,32,33,34] go into further detail regarding the ecological, engineering and IT-related research that was achieved during this period of SEMAT.

However, at the time SEMAT was conceived, there was still much pull amongst the research community drawing the project in multiple directions and adding “*bloated functionality*” to the system. Here, bloated functionality refers to features that are peripheral to the basic functional requirements of the system or excess to the actual needs of the system end-user. This bloated functionality was also often research driven, pursuing a dedicated thread of its own which did not contribute to creating an immediately operational system serving the needs of environmental monitoring. Based on a review of the goals outlined in Trevathan et al. [8], the post deployment analysis for the Deception Bay and Heron Island deployments and the advancements in technology since Trevathan et al. [8] was published, we have refined the goals for SEMAT to be the following:

1. *Application-driven deployments and reporting*—Data is collected for scientific study purposes and for client-oriented needs such as validation of compliances or standards in water quality; not just for the sake of collecting data. Deployments and data collection are driven by an underlying application where the data informs the user and provides measures of condition or performance relevant to the end-user;
2. *Low Cost—*By utilising suitable “*off-the-shelf*” elements and an innovative approach to device production, the aim is to deliver a robust sensor network system which is significantly under 40% of the cost of similar existing systems on the market;
3. *Minimal deployment expertise*—A buoy must be easily deployable without requiring any onsite configuration and specialist equipment and can ideally be done by a single person (e.g., the buoy can be deployed and its operation immediately checked via a smart phone);
4. *The ability to adapt and evolve*—The SEMAT system design must be able to evolve with enhancements in technology and community needs. Through modularisation and an adaptable architecture, the core system can be modified ongoing to meet user requirements.
5. *Remote interface, data management and analysis tools*—The SEMAT system needs to provide a user interface, backend data management and set of simple, yet powerful tools for an end user to view and manipulate the data and monitor/manage the system;
6. *Robustness/durability*—A buoy needs to be robust enough to withstand harsh conditions without failures over an extended period of deployment (e.g., heavy rain, waves, strong current, hail, boat strikes, fish/bird attacks, bio-fouling, submersion, etc.);
7. *Transferability/sustainability of product*—The SEMAT development and production model must continue to move forward rather than stop after a specific project/deployment, or be larger than a single individual; and
8. *Small environmental footprint—*The buoy must be constructed using the minimal possible resources and ideally most components can be recycled/reused once a deployment is over. A buoy must not negatively impact on the environment around it in which the buoy is deployed (e.g., toxic glues/paints or materials being left behind post deployment).

Several goals outlined in Trevathan et al. [8] have been removed as they are deemed peripheral and/or too superfluous to pursue with limited resources and personnel. SEMAT aims to focus on establishing a practical and sustainable environmental monitoring system that serves the purpose of providing data for direct application. There is no aspiration to solve all of the more global technical challenges facing conceivable WSNs, nor the futuristic aspirations of environmental monitoring technological research beyond those solved as the system is evolved and tested.

Given that sensor buoys will be deployed in remote locations, the amount of time servicing them in the field must be kept to a minimum. Therefore, it is desirable to keep a buoy as simple as possible. “What can go wrong, will go wrong.” As such, SEMAT aims to follow the “KISS” principle—*Keep It Simple and Stupid*. This means that the technical and functional complexity of a buoy must be kept as minimal as possible to limit the potential for errors. Instead, the “smart stuff” occurs at the server level, which is accessible and scalable; the expense, logistics and risks of retrieving and servicing a malfunctioning buoy exceeds that of having a programmer fix an issue on a server.

In-line with the SEMAT philosophy, the system should be inexpensive so that deploying numerous devices can cover a large spatial area. Furthermore, the system is capable of operating for extended deployments (≥12 months) with maintenance cycles in the order of three months to address issues such as bio fouling. Purposely the SEMAT system is intended for operation under the rigors of near-shore marine and estuarine ecosystems, as well as rivers, creeks, wetlands and constructed water bodies. 

### 2.3. The Need for an Open Standard for Constructing Environmental Monitoring Systems

The SEMAT project has necessarily required expertise from multiple disciplines including:

- *Data Application and Modelling* (environmental and mathematical);
- *Application programming* (user interface, data and content management, algorithms);
- *Systems programming* (networking/communications, hardware-level details);
- *Electrical Engineering*/*Physics* (electronics and sensor development); and
- *Field/Deployment* (experience in marine hardening systems, testing and deployment).

Only through this multidisciplinary approach have we been able to match end-user requirements with technical design and the required functionality.

In order to address Design Goal 7 (Section 2.2), the paradigm for creating environmental monitoring systems must be inverted. To date, mainstream approaches taken by scientific companies or large funded government agencies have not delivered the desired outcomes or a sustainable way of conducting environmental monitoring; especially where the end-users operate under constrained funding and human resource conditions. At the same time, no one company can be expected to cross all aspects. As such, a community-driven approach would instead seem more applicable and more likely to deliver an outcome appropriate to and easily assimilated by end-users.

Consider the approach for developing 3D printing technology. Historically, 3D printers were expensive and required highly trained staff to operate them. The RepRap (http://reprap.org/) project introduced a new paradigm whereby an open source design model was proposed to allow everyone to construct a 3D printer (even using 3D printed parts). People could innovate around the RepRap model and the community drove the development. Now 3D printers can be constructed at low-cost (e.g., $64) with only limited technical expertise required.

In this paper, we are proposing a similar “Open Design” based on SEMAT (similar to the initiative by Sadler et al. [9]). Insights from the RepRap project are relevant to solving the challenges of driving down the costs of creating environmental monitoring equipment. Accordingly, the SEMAT project seeks to allow the community to reproduce and innovate around the core design so that end-users obtain a system relevant to their specific needs but compliant with a standard suited to comparisons and exchanges with other similar systems (Design Goal 4, Section 2.2). 

Fundamental to the SEMAT approach is the modularization of the system. Beyond the electronic and sensor design elements, the monitoring system needs to be easily serviced and maintained under a wide range of operational conditions. In this context, a modular approach to the design is highly preferable as it allows the operator to inspect, service, or replace components with little to no risk of damage or degradation due to field conditions. 

### 2.4. Engaging the Community in Production and Education through Social Enterprise

A *social enterprise* is an organization that addresses a basic unmet need or solves a social problem through a market-driven approach [35]. In recent years, traditional non-profits have become more entrepreneurial and interested in generating earned revenue to supplement charitable contributions. Furthermore, traditional businesses have begun to integrate greater levels of social responsibility and sustainability into their operations. The growth of social enterprise is a reflection of this convergence and helps fill the void between traditional approaches that have focused solely on creating *either* social impact *or* financial returns.

Substation33 (substation33.com.au) is a social enterprise located in Logan Australia. Substation33 was originally established as an electronic waste recycling social enterprise business in an effort to reduce the amount of electronic componentry being dumped as landfill. Substation33′s primary charter is to connect with people marginalized from mainstream employment for a variety of reasons (such as long-term unemployment, physical or other disability, early school leavers or students at risk of disengaging from school). These people are then mentored by community leaders and are engaged in innovation projects that promote sustainable environmental practices [36].

As previously mentioned, this paper seeks to formalize SEMAT as an open standard to tap into the ideas of the scientific, industrial and hobbyist communities to drive the innovation behind environmental monitoring technologies. As such, social enterprise is one mechanism towards this goal. In 2017, SEMAT formed a relationship with Substation33, whereby Substation33 is engaged in the production of SEMAT devices using up-cycled e-waste (Design Goal 8, Section 2.2). In this way, the SEMAT initiative also brings the benefits of social enterprise to community through education and reduced construction costs. To our knowledge this is the first time such a development and production model has been proposed for improving environmental monitoring outcomes and is the subject of an ensuing publication.

## 3. An Open Standard System for Low-Cost, Remote Aquatic Environmental Monitoring

### 3.1. System Architecture

SEMAT has taken a hierarchical approach to sensor network architecture. The network architecture presented in Trevathan et al. [7,8] consisted of the individual field units transmitting to a land mounted base station and then through the Internet via a 3G modem to a back-end server. This avoided the complexity associated with multi-hop network architectures.

While this approach has some merits, experience in early deployments highlighted its drawbacks. For example, in this configuration all buoys are logically grouped and are dependent on a specific base station. However, the base stations reliability represents a central point for failure for the whole system. Additionally, base stations must be repeated to extend the geographic coverage adding significantly to the system’s expense and level of complexity.

It is noted that some local government bodies in Australia are experimenting with IoT providers for establishing low power wide area networks (e.g., Taggle (www.taggle.com.au) and Sigfox (www.sigfox.com)). However, this approach requires a council to be liable for all costs associated with installing and maintaining these base stations. Base stations must be repeated (every 10–15 km) and direct line of sight is usually required. Furthermore, such systems have limited spatial coverage when it comes to monitoring rural or off-shore areas.

**Figure 1:**
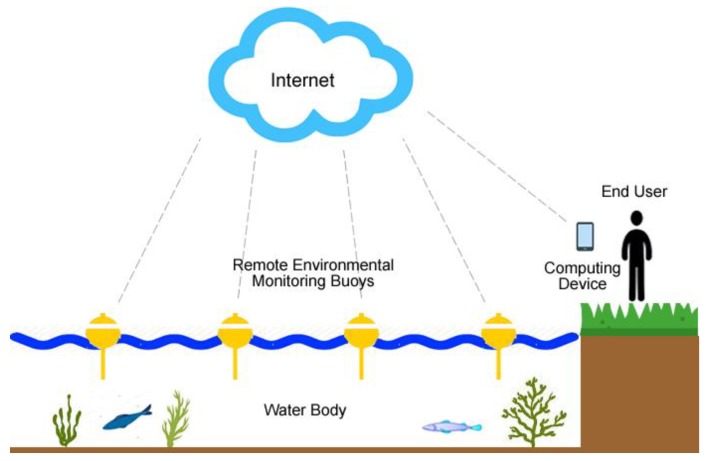
The Smart Environmental Monitoring and Assessment Technologies (SEMAT) Aquatic Sensor Network Architecture including data access via mobile devices.

Due to the aforementioned limitations, SEMAT utilizes existing network infrastructure as maintained by the telecommunications companies (see Figure 1). This greatly reduces both the cost and infrastructure required, whilst providing a greater spatial coverage and reliability. Increasingly, GSM (*Global System for Mobile Communications*) technology is at a stage whereby an individual buoy is able to directly communicate with the national network; even in most regional locations in many countries. The only trade-off with this approach is an ongoing subscription to a service via a SIM (*Subscriber Identity Module*) card. However, the cost of this approach is significantly lower than the purchase, installation and maintenance of an independent communications network. Where this connectivity is not possible, the SEMAT standard also allows any type of communication method to be used based on the user’s requirements (e.g., Zigbee, LoRa, WiFi).

### 3.2. Physical Components of a SEMAT Buoy

**Figure 2:**
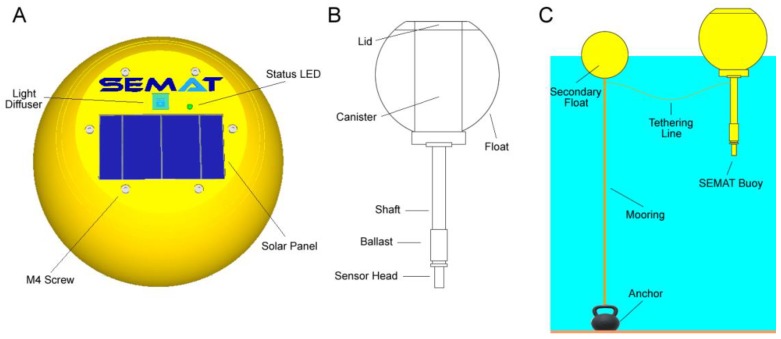
(**A**) SEMAT Buoy (Top View); (**B**) Buoy Cross Section; (**C**) Anchor and Mooring System.

SEMAT has experimented with many buoy designs with varying results. Figure 2 shows the latest SEMAT buoy. The physical components of a buoy consist of the following main parts:

- *Float*—provides the main buoyancy and physical structure of the buoy;
- *Lid*—seals the canister and houses above water sensors, solar panels and status LED;
- *Canister*—houses the internal electronics components and provides additional buoyancy;
- *Shaft*—extends from the canister below water to connect the sensor head;
- *Ballast*—provides weighting to ensure the buoy maintains upright positioning in the water;
- *Sensor Head*—houses the underwater sensors; and
- *Mooring and Anchor*—tethers the buoy in position in a water body.

The main buoyancy element is a modified 250 mm styrene float. This provides the buoyancy required for the canister and associated componentry. A 91 mm cylindrical cut is made through the centre of the buoy to insert the canister and a 40 mm chord is cut from the top of the buoy perpendicular to the cylindrical cut to facilitate the placement of the lid. Primer and weather proof paint were used to colour the float and provide a thin water proof skin.

The lid is a 3D printed design that is 190 mm at its base matching the curved surface of the top of the float. The lid incorporates the solar panel, with two penetrations underneath for the wiring to reach the canister. The diffuser and light sensor for surface light readings is also integrated into the buoy lid. A tri-colour status LED is also inserted in the lid and allows the state/operation of the buoy to be observed externally in the field (i.e., Green—sleep, Blue—awake/transmitting, Red—error). The lid is fixed to an underlying flange via six M4 × 16 mm stainless steel 316 screws that compress the lid onto an O-ring seal with the underlying canister.

The canister is a 90 mm PVC pipe sealed on the bottom with a standard PVC end-cap using traditional PVC primer and glue. A 3D printed flange is adhered to the top in the same manner to facilitate connection with the lid. As noted, the flange contains a countersunk O-ring for a water tight seal (coated with silicone grease). A standard irrigation quality flange and pipe connection (20 mm diameter) provide the watertight connection between the canister electronics and the sensor set attached at the end of the 0.5 m shaft. The plastic threads are sealed using PTFE thread tape.

The shaft is a ¾”/½” × 300 mm polypropylene riser with a male (top) and female (bottom) BSP thread. The shaft hosts an electrical cable (Cat5) that connects the sensor head to the electronics in the canister. The shaft also contains a rod of metal that serves as a ballast bar so that the buoy maintains an upright position in varying wind and wave conditions. Having the ballast within the shaft prevents any corrosion with chemical reactions from the water. The ballast can also be recycled for reuse with other buoys.

The sensor head is a PCB containing the sensors. The sensor head connects to the shaft via a male BSP thread. (The sensor head is described in further detail in Section 3.3.5).

The mooring system shown in Figure 2 consists of a secondary float connected to the SEMAT buoy by a tethering line. The secondary float is attached to an anchor via a mooring. For the deployments outlined in this paper, a plastic coated 8 kg weight was used as an anchor. The weight has an in-built handle which provided an attachment point for the mooring line and also aided in deployment and retrieval (a range of low-cost solutions are available for use as an anchor). By tethering the sensor buoy to a separately moored buoy there was no interference between the anchor line and the sensors. Also, the SEMAT buoy was able to remain upright as the ballast and tether line counter balanced the influence of water currents and wind. This also reduced the possible interference of additional fouling from having an anchor line in close proximity to the sensors. Other mooring systems may be more applicable in different situations and conditions.

Note that this physical buoy design represents one of many options we are experimenting with. A newer, more compact and sturdier design with a twist on lid is being tested at the time of writing this paper. The overall design consideration is to make the buoy as low-cost as possible, using readily available materials and reducing the amount of labour required for construction, deployment and maintenance.

### 3.3. Electronics Subsystems of a SEMAT Buoy

A systems approach has been taken in the development of the SEMAT buoy electronics. This allows the complexity to be managed and related functionality, so it can be grouped according to a particular subsystem. Table 3 shows the subsystems for a SEMAT buoy. The following sections outline the functional requirements for each of these subsystems, the current technologies employed and the rationale for the design approach.

**Figure 3:**
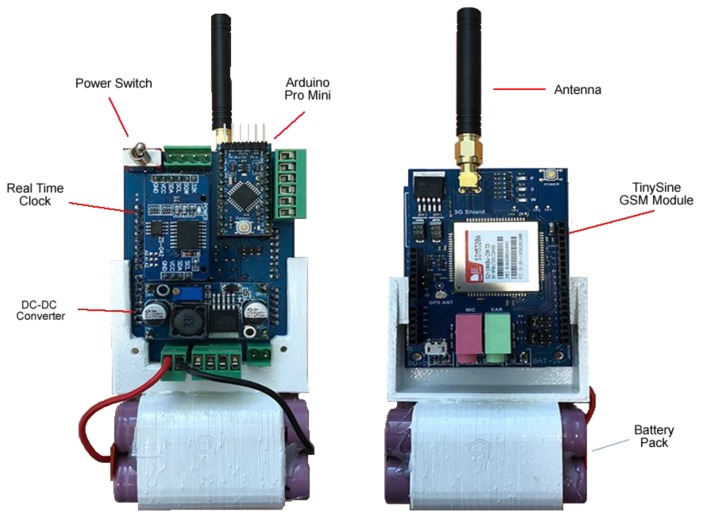
SEMAT Electronics.

Figure 3 illustrates the internal electronics for a SEMAT buoy (front and back). Each of the major components is described in each respective section below.

#### 3.3.1. Microcontroller Subsystem

The microcontroller subsystem is essentially the brain of the buoy. Once awake, the microcontroller initializes all system objects (devices), takes the sensor readings, transmits the readings and then initiates the next sleep cycle. As previously mentioned, the amount of functionality on the buoy needs to be kept to a minimum to limit the number of issues that can occur. The code on the microcontroller must always have an end state to ensure that no logic path can result in an infinite loop or timeout, which will run down the battery (by preventing the system from entering a sleep state). Section 3.3.4 outlines in detail the various system states.

We chose the Arduino microcontroller primarily due to its simplicity and widely known properties. While other more powerful microcontroller platforms such as Raspberry Pi can be used, these tend to consume more power—which is a scarce resource. Other ultra-low power draw microcontrollers tend to be more complicated to operate and require intricate memory management manipulation. We used an Arduino Pro Mini ATmega328P (5 v, 16 MHz) for the deployments outlined in this paper. This board was chosen due to its compact form factor and low power consumption.

#### 3.3.2. Power Subsystem

Arduino and associated sensors offer two voltage logic levels: 3.3 v or 5 v reflecting the shift in hobby electronics towards 3.3 v logic in order to reduce battery sizes; the 3.3 v also lends itself naturally to LiPo and Li-ion voltage levels (i.e., around 3.7 v). Whilst trials were undertaken using 3.3 v logic, it became apparent that many sensors and other devices, such as servos, require 5 v logic in order to operate. Therefore, the decision was made to make the system operate at 5 v to avoid having to complicate the system using logic level shifting electronics.

In light of this and the gains made through the use of up-cycled LiPo or Li-ion batteries (from social enterprise collaboration), it was necessary to utilize a DC-DC converter to achieve 5 v from the 3.7 v to 4.2 v available. This component serves two purposes. Firstly, the DC-DC converter takes the battery voltage and boosts it up to the required 5 v. Secondly, the DC-DC converter creates a smooth 5 v signal regardless of the battery voltage and potential current spikes on the system. The current design uses four recycled AA Li-Ion batteries operating at approximately 3.7 v–4.2 v providing 8000 mAh. 

As the system does not need to sample continuously, duty cycling is desirable as a way to conserve battery power. While there are ways to place an Arduino into a quiescent state through various sleep options, this does not power down all of the peripheral electronics (e.g., the DC-DC converter, various sensors, etc.). As such there is a significant continual power drain on the system. To overcome this issue, an additional timing circuit was installed to actively turn off all system devices. Our design uses a MOSFET N-Channel and JK flip-flop in conjunction with the system’s clock (described in Section 3.3.4). The Arduino sets an alarm on the clock to wake up in 15 min and turns on the green sleep status light by sending a voltage high signal on the appropriate pin of the status LED. This triggers the flip-flop to change state and the MOSFET powers the system down. When the real time clock alarm signal is detected by a rising edge triggered on its SQW pin, the flip-flop changes state again and the MOSFET powers the system up to take the next round of sensor samples. This process repeats indefinitely.

#### 3.3.3. Solar Charging Subsystem

A 6 v 1 W solar panel is used to recharge the batteries. A CN3083 SOP8 high efficiency solar charging circuit chip is required to convert 6 v down to the appropriate level for the Li-ion battery pack. Optimally the batteries operate in the range of 3.7 v–4.2 v. Overcharge or extreme discharge of the battery pack is undesirable. So critical is the role of the battery in the system that voltage is measured via one of the Arduino’s analogue pins and transmitted back to the server along with the other sensor samples. Creative ways to allow the buoy to adjust its sampling time to improve battery life can be used when low voltage conditions are present. For example, when a low voltage condition is detected, the system will sample less often until the battery voltage recharges to an acceptable level. Additional circuitry (a voltage divider and MOSFET) was added to prevent overcharge and discharge on each battery cell. 

#### 3.3.4. Timing Subsystem

System timing is critical for determining when to take samples and correctly time stamping the samples. There are three sources of timing for the system:

- A *Real Time Clock* (RTC) located on the buoy;
- Obtaining the time from the GSM network; and
- The time on the backend server database when a transmission is received.

The RTC provides a way to keep track of the time (either 24-h or 12-h format) so that sensor readings can be time stamped. SEMAT employs the DS3231 RTC by Texas Instruments. The RTC contains a CR2032 coin-cell battery to allow it to keep track of the current time. The RTC is independent of the Arduino. The battery cell can cast for up to 5 years. The time set in the RTC is synchronized with the computer that loads the script onto the Arduino. However, over time we observed that there is quite significant time drift and variation in alarm timing between different DS3231 RTCs. If a buoy is relied on to timestamp its own sensor samples, then there is the definite likelihood that all deployed buoys will not be sampling at a common time in synchrony. As such, apart from setting an alarm to control duty cycling, the RTC should not be relied upon for time stamping sensor samples.

A common time can be obtained via the GSM network. Here the time of transmission can be logged but the RTC cannot be easily updated to resynchronize all buoys. Ultimately, the easiest approach is to have the backend server log the time a sample is received from each buoy uses the database’s common timestamp. This removes the responsibility of time stamping from the buoys (and the need for the CR2032 battery) but does not fix issues of buoy synchronization. Experience indicates that on a 15-min sample interval, all buoys will transmit within 7 ½ minutes of each other. Generalized, this means that for an *n*-minute sample interval, all buoys are synchronized to transmit within *n*/2 min of each other.

**Figure 4:**
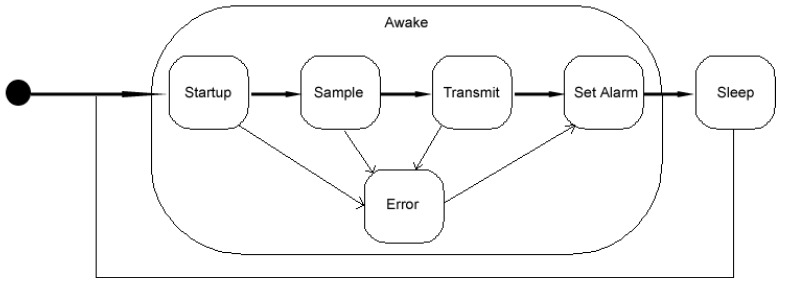
SEMAT Buoy States.

Figure 4 illustrates the various states of the buoy based on the timing. Upon initially turning on the buoy by connecting it to the power system, the buoy will cycle between two major states—*Awake* or *Sleep*. When an Awake state is triggered, the microcontroller subsystem will enter into a *Startup* state and initialize all system objects in memory and check all attached hardware. Next, the system will take sensor samples in the *Sample* state, then enters in to the *Transmit* state to send the readings to the web server. If at any stage an error occurs, the system will momentarily enter the *Error* state and notify the user either via a red status LED indicator, transmit status information and write an error to the Arduino’s EEPROM. Finally, the system will enter a *Set Alarm* state to set the alarm on the RTC. The system then enters into the Sleep state until the alarm wakes the system up again and the process repeats.

#### 3.3.5. Sensor Subsystem

The core electronic sensors on a SEMAT buoy included: Temperature; *Photosynthetically Active Radiation* (PAR) (equivalent); Turbidity; and Depth.

We have primarily focused on these parameters as they are fundamental to water quality management. Based on these parameters, other readings from chemical sensors can be approximated. The problem with chemical sensors (apart from expense) is their long-term instability and need for regular maintenance in order to maintain accuracy. Most sensors need to take water samples, mix chemicals and then interpret colours or other types of readings. The membranes in such devices are usually the first point of failure. 

Sensors must be approximately 0.5 m below the water surface. The reason being that surface-only readings are insufficient. The readings must be taken deep enough in the water column to ensure proper mixing and hydrological processes to occur. PAR is taken at the surface and 0.5 m below the surface to determine the *light attenuation curve* (i.e., the change in light between the surface and underwater).

For the deployments outlined in this paper we used the Adafruit MCP9808 Precision I2C Temperature Sensor (https://www.adafruit.com/product/1782). This sensor can operate between −40 °C to +125 °C with accuracy of +-0.25 °C. Less expensive temperature sensors such as the Maxim DS18B20 are also sufficient for the task.

With regard to light, the most widely useful measurement type in environmental management is that of PAR which spans wavelengths from 400 to 700 nanometres. Rather than use an expensive proprietary PAR sensor, we employed the Adafruit TSL2591 High Dynamic Digital Light Sensor (https://www.adafruit.com/product/439). This sensor can detect light intensities from 188 µLux to 88,000 Lux and is able to detect light across the spectrum covered by PAR. As such, we are able to calibrate this sensor against a PAR sensor to provide a PAR equivalent. Slightly less expensive versions of this sensor can be sourced on eBay. However, in our experience most alternatives were hard wired to a single I^2^C address, whereas the Adafruit TSL2591 allows up to three I^2^C addresses.

To measure water clarity, our turbidity sensor was based on components salvaged from the Gravity Analog Turbidity Sensor (https://www.dfrobot.com/product-1394.html). This sensor is able to detect suspended particles in the water by measuring the light transmittance and scattering rate which changes with the amount of total suspended solids in the water. Once calibrated, this sensor is able to provide a derived *Nephelometric Turbidity Unit* (NTU) value. We used the Gravity’s existing infrared sender and receiver and some of its physical housing. We then added our own low pass filter circuit, determined the optimal operating voltage for sensing and calibrated the modified turbidity sensor against a highly accurate certified turbidity sensor and calibration standards

Normally, water depth measurement is performed by a transducer in conjunction with some marine computing device. Anything electronic that has to be near water always comes with a higher “marine grade” price. The proposed solution was to find something low cost and already in abundance. While not the most scientific application, a “waterproof” reversing sensor that is normally installed on the bumper of a car was trialled for suitability as a depth sounder. 

Along with its resistance to being wet, key elements that made this a promising solution within an aquatic senor network was the potential distance it could reach. Since the density of water is more than 4 times greater than air, the sounding signal could potentially reach as far as 16 m before timing out. Coincidentally, the frequency at which a purpose-built depth sounder uses to find the waters bed is about 50 kHz; which is rather close to that of the reversing sensor. The reverse sensor kit used is a JSN-SR04T, operating at an ultrasonic frequency of 40 kHz. Used in its intended environment, it has a minimum range of 200 mm, a maximum range of 4000 mm and has an accuracy of ±50 mm. An air test showed a 6% error tolerance over its entire range and the tolerance was found to be less than 3% after the first meter of measurement.

The performance tests underwater showed that there is little to no impact on the effectiveness of the reverse sensor when the ultrasonic frequency was traveling through a different medium; water. The raw value provided by the hardware was converted into a standard measurement and calibrated to the travel medium. This sensor was deployed in water for several days with no further waterproofing and there was no degradation found in its performance, indicating long term durability.

Other potential Arduino-based sensors that can be added to the system include: Salinity/Conductivity; Dissolved Oxygen; and pH. These sensors are available from Atlas Scientific (www.atlas-scientific.com). (Note that these latter sensors are not used in the deployments outlined in this paper.)

**Figure 5:**
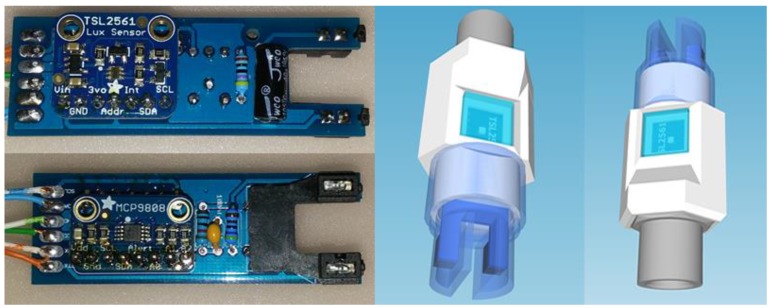
Sensor Head.

The underwater sensors were consolidated into a single sensor head (Figure 5). This is a custom designed PCB that provides all of the appropriate electrical pathways and turbidity sensor circuitry. The sensor head is encased in a 3D printed enclosure and contains a male BSP thread to allow it to be attached to the shaft of the buoy.

***-Sensor Calibration-***

Inexpensive sensors need to be calibrated against more expensive, highly accurate sensors prior to deployment or by means of direct physicochemical methods.

To approximate realistic operational conditions, the light sensors were calibrated against a certified and calibrated LiCor^®^ light meter using a dedicated LI-190R Quantum PAR sensor head. The SEMAT light sensor was simultaneously exposed to the same range of calibration light regimes as the LiCor^®^ device to produce a response and calibration curve. Different light intensities and frequency ranges were established using standard light frequency filters. This method allowed for rapid checking of the sensors as they are produced and also provides a means of field-based checking of sensor performance over time.

**Figure 6:**
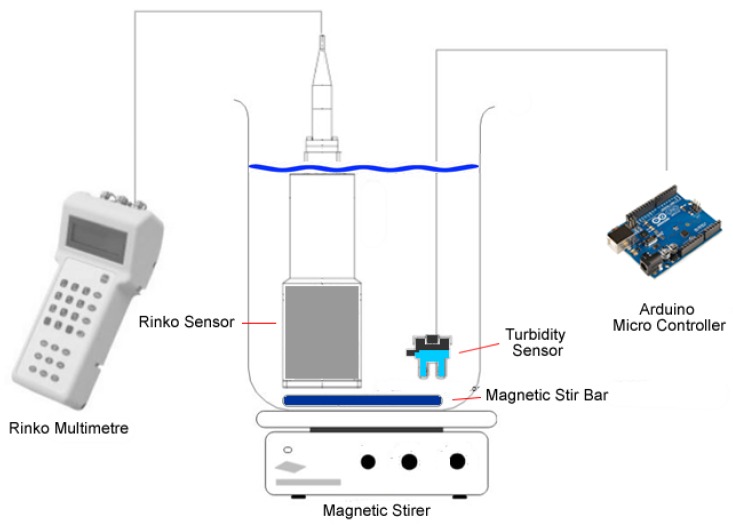
Turbidity Calibration Setup.

As with light measurement calibration, the turbidity sensors were calibrated against a certified and calibrated ALEC-Rinko^®^ turbidity sensor. As illustrated in Figure 6, the SEMAT turbidity sensor was simultaneously exposed to the same range of calibration turbidity regimes as the ALEC-Rinko^®^ device to produce a response and calibration curve. Different turbidity level intensities were established using turbidity standards. Again, this method allowed for rapid checking of the sensors as they are produced and for field-based checking of sensor performance over time.

#### 3.3.6. Communications Subsystem

As noted previously, the SEMAT standard allows for any type of communication method to be used based on the user’s requirements (e.g., Zigbee, LoRa, WiFi, etc.). Current deployments utilize the existing GSM network to take advantage of existing infrastructure and the cost savings this provides in terms of removing the need for additional infrastructure and maintenance. 

Initial trials utilized the Adafruit FONA 808 MiniGSM + GPS (https://www.adafruit.com/product/2542). This breakout board comes with full data support for HTTP and also GPS. However, while this version of FONA was sufficient for the task, it only provides frequencies for operating on the 2G network. In countries such as Australia, the 2G network has now been turned off and no longer supported by all telecommunications companies. Notably, however, a number of developing nations will continue to support 2G for several more years making the FONA 808 a viable intermediate option. 

More recently Adafruit has released the FONA 3G Cellular + GSM (https://learn.adafruit.com/adafruit-fona-3g-cellular-gps-breakout/overview) using the SIM5320 chip. This module works on all of the 3G frequencies supported by the Australian telecommunications companies. TinySine (https://www.tinyosshop.com) has created a less expensive version of the FONA 3G. As stated on the Adafruit website, there is limited support for the data component of their SIM5320 chip. However, at the time of writing, to our knowledge we are the first to reliably send data using the HTTP protocol via 3G. 

#### 3.3.7. Cleaning Subsystem

As the PAR lens and turbidity sensor are subject to fowling, a mechanism is required for cleaning [37]. Several wiper arm approaches can be used depending on the type of fouling. A servo is timed to control the wiper arm to periodically wipe across the surface of the lens. There are also chemical-based approaches including anti-fouling paints and surface applied repellents. For the deployments outlined in this paper we did not employ any cleaning mechanism other than periodic manual cleaning. We intend to pursue this in future work.

### 3.4. User Interface

**Figure 7:**
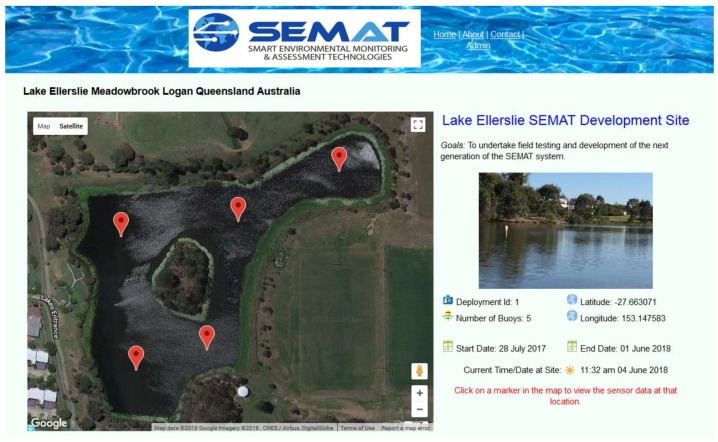
Web-Based User Interface.

**Figure 8:**
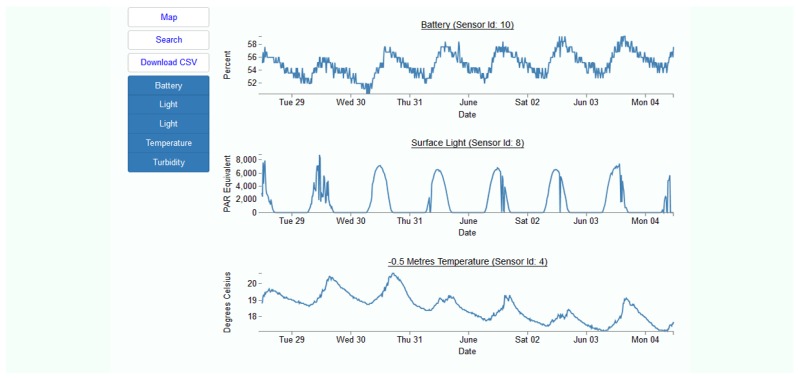
Charted Data from a Buoy.

As presented in Figure 7, a web-based user interface was developed to present data and to allow the end-user to view deployment/sensor node locations on a Google^®^ Map (Design Goal 5, Section 2.2). An individual sensor node can be selected and its corresponding sensors are displayed. The user then has the option of charting and/or downloading any sensor’s data (Figure 8). Information is also available to the user about power supply status and alerts are issued if data is incomplete or a buoy fails to transmit. A user is also able to set flags to be alerted when certain environmental parameters change suddenly, reach a threshold value, or a combination of parameters indicate an imminent change in environmental conditions.

**Figure 9:**
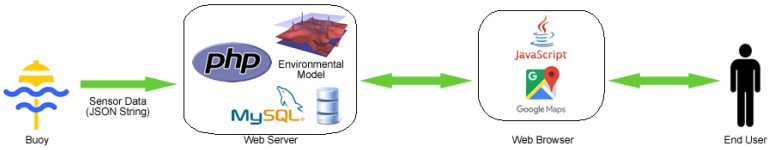
Software Architecture.

Figure 9 summarizes the SEMAT software architecture. A buoy transmits its data as a JSON string to the web server. The web server uses PHP as the scripting language to process and store the incoming data into the MySQL database. An environmental model consumes the data. A user then views the data via a web browser that uses a combination of JavaScript and Google Maps. The full software design will be documented in future work.

## 4. Environmental Study and Field Test Deployments

### 4.1. Lake Ellerslie

**Figure 10:**
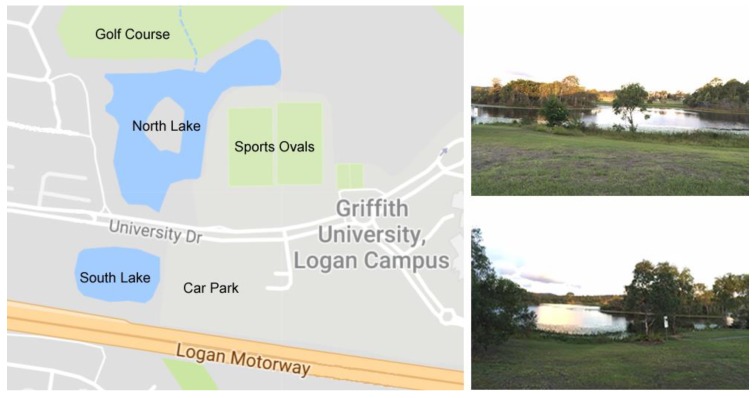
Lake Ellerslie Griffith University Logan Campus.

Lake Ellerslie is an artificial lake that is part of Griffith University’s Logan Campus (Figure 10). The lake consists of two parts—the north and south lakes. The lake depth varies between 2 and 6 m across different areas. Both components of the lake are connected via a conduit under an intersecting road (i.e., University Drive) and collectively serve to detain storm water flows from the adjacent motorway as well as to assist in local flood mitigation. The two lakes become detached during long periods of low rainfall such that the respective water bodies then have no interaction. Also, the north lake drains via a waterway through a golf course to Slacks Creek when water levels exceed drain height. 

As indicated, the south lake receives input from the Logan Motorway and adjacent car park whilst the north lake is impacted by nutrient run-off from the sports fields, residential premises and golf course. The lake system is only flushed during continuous or high intensity rainfall events so that aspects such as water depth are important considerations for local waterways managers. In this context, the lake system reflects a typical situation for many peri-urban water bodies in Australia and similar locations internationally. 

From a waterway’s management perspective, Lake Ellerslie serves two purposes:

1. To filter sediment and contaminants from the motorway and surrounds; and
2. To relieve and redirect flooding from Slacks Creek.

In the first instance, the south lake captures the immediate pollutants from the Logan motorway and car park via storm water drains. This includes all sorts of car fluids, road debris and rubbish. There are a series of sediment catchment sites at the entry points of these inputs with the majority of the initial non-dissolved contaminants and sediment settling or captured in the south lake. When the two lakes are connected during periods of high or extended rainfall, overflow from the south lake feeds into the northern lake taking materials/contaminants with it. Should this condition persist, then overflow continues out into the associated Slacks Creek and natural environment. 

Notably, should a significant flood event occur, waters can flow in the reverse direction due to the higher flows and tidal influence forcing waters from Slacks Creek up into the catchment. This has occurred on numerous occasions over the last 20 years. In view of this and the general performance of the lake system, it represents a local focal point for understanding potential issues with water contamination and flood mitigation.

In addition, the lake supports significant fish, turtle and wetland bird populations. The centre island, for example, is a roosting sanctuary for several varieties of indigenous birds, including large numbers of ibis who contribute large volumes of faecal material to the lake waters. Because of this, the algal growth it has encouraged and the intermittent detection of significant *Escherichia coli* (*E. coli*) numbers in lake waters, Lake Ellerslie has signage preventing human use of the lake. Any use of the lake water for local irrigation requires the water to be chlorinated first.

Given this history and the local management situation, Lake Ellerslie presents an ideal and locally relevant site for test deployments and systems testing. In addition, the lake has the following attributes:

- *Accessibility/Convenience*—The lake is part of Griffith University Logan Campus. No permits (beyond university approval) are required.
- *3G Coverage*—The lake is within range of 3G, therefore remote monitoring of the lake can occur without using additional network infrastructure.
- *Low-Cost*—As no travel is involved, it is relatively inexpensive to deploy and monitor SEMAT buoys.
- *Unstudied—*Since the lake was constructed throughout 1995–2002, there have been no comprehensive documented studies of the site. (Except for an out dated Masters research dissertation of the south lake from 1999 [38].)

### 4.2. Deployment and Environmental Study Results

SEMAT was awarded a Logan City Council EnviroGrant to undertake a study of Lake Ellerslie from July 2017 to June 2018. During this period, the project sought to achieve the SEMAT goals as outlined in Section 2.2.

**Deployment 1—Systems Test**

The first deployment occurred over five weeks throughout August 2017 involving five buoys. This was largely a systems test using a foam body and flexi-shaft as a precursor to the design summarized in Section 3. The buoys took light readings (water surface and at 0.5 m depth), temperature (0.5 m depth), turbidity (0.5 m depth) and also measured system power status. Readings were transmitted every 15 min via the now obsolete 2G network. The light and turbidity sensors were not calibrated but used to test systems integration, power management and communication strategies. Throughout this deployment the buoys went into a sleep state in between readings/transmissions—but not all electronics could be powered down completely.

**Deployment 2—Production Line and Calibration Test**

**Figure 11:**
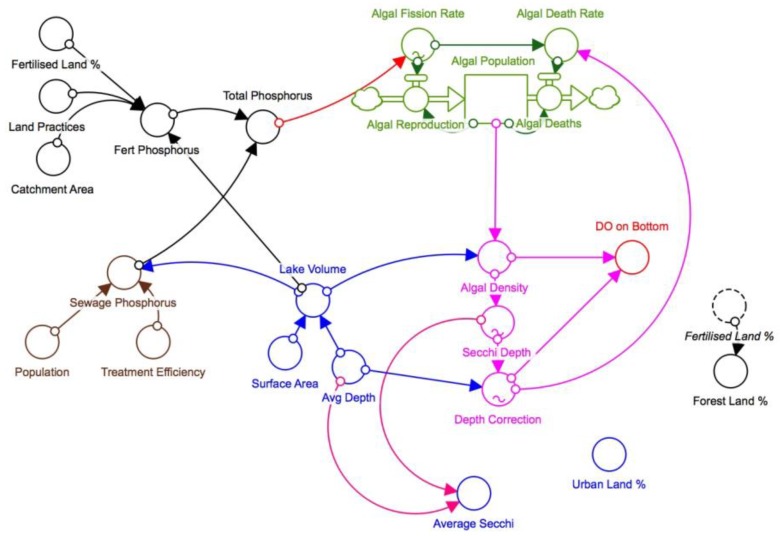
Lake Ellerslie Dynamic Systems Model.

The second deployment occurred over 10 weeks from March to June 2018. In addition to further systems testing, this deployment involved significantly enhanced electronics and physical construction. The actual buoy design was in line with the system and properties outlined in this paper using the revised integrated sensor head design. The sensors were also calibrated to allow for focused accuracy and performance assessment. A purpose designed printed circuit board was also utilized in this system to reduce the complexity of the system. In addition, an improved power management methodology was developed utilizing a smaller battery pack and the system was upgraded to the 3G network and trialled a new sensor head design. A dynamic systems model was created of the lake (see Figure 11). Turbidity data from the deployment was input into the dynamic systems model of the lake and used to inform and validate the model to improve its predictive capability. 

A high school innovation challenge was also undertaken in conjunction with the 2nd deployment. This involved 78 Year 10 students who were tasked with visualizing the data coming from the sensor network in a more meaningful and intuitive manner. The purpose of this innovation challenge was to promote community engagement and ownership of environmental management problems at the local level. Some of the ideas put forward will be adopted by the SEMAT system and documented in future publications.

### 4.3. Evaluation of the Deployments and System Against SEMAT’s Revised Design Goals

This section assesses how the system deployed in the Lake Ellerslie study performed against the criteria for SEMAT’s design goals as outlined in Section 2.2.

1. *Application-driven deployments and reporting*—The application was to investigate the function of a lake and assess the keystone influences on lake water quality. In light of the very limited historic monitoring for lake waters, the deployment was successful in describing water quality responses to multiple weather events and to changes in human inputs over the study period.

2. *Low-cost*—Table 4 contrasts potentially similar research and commercially available buoys against SEMAT. The final material cost for the SEMAT buoy is approximately $154 USD. The approximate cost of the recycled components if purchased new would be around $50. A monthly subscription to a telecommunications company for SIM data is $5 per month. We used a cloud-based server for our web hosting which costs $5.50 per month. During the deployment, we were able to assess the sensitivity and accuracy of the applied sensors over varying water and operational conditions to show that a low-cost approach to sensor selection is indeed sufficient for the task of scientific monitoring.

The cost for SEMAT does not take into account the research and development effort or the logistical costs of deployment. However, unlike a commercial venture, development outlays do not need to be recovered. This illustrates the importance and merit of using SEMAT’s approach to research and development using a combination of universities and social enterprise to distribute expenses by employing resources that are part of ordinary work-related duties. A more detailed analysis of the SEMAT production methodology is the subject of an ensuing publication. (Note that a cost comparison with other research buoys is difficult as prices are generally not made available.)

3. *Minimal deployment expertise*—The proposed design allowed for easy deployment and recovery of buoys. Once a buoy was assembled in a controlled environment, no on-site configuration was required during deployment. No special cranes or equipment were necessary (beyond a boat) for deployment in contrast to other commercial and research proposals. No diving was necessary. Retrieval of the buoys post deployment only required detaching them from their moorings (note that the moorings were left in situ for future deployments).

4. *The ability to adapt and evolve*—The modular and simple design allowed individual componentry to be updated based on technological advancements. As the design is based on open source technologies, it was not restricted to any proprietary product. An example of this flexibility was evidenced by the easy ability for the system to change from 2G to 3G for deployment 2.

5. *Remote interface, data management and analysis tools*—We provided a web-based interface and back-end data management system based on openly available software. We also incorporated a dynamic systems model for interpretation of the data. The aforementioned high school challenge also allowed us to engage the community in the visualization and interpretation of the data to help educate the students about how their actions impact upon water systems.

6. *Robustness*—During deployment 1, one buoy suffered water ingress four days after deployment due to an incorrectly fitted O-ring around the lid. A second buoy highlighted the potential for interference from externalities such as water birds and failed after 28 days. Notably, significant and intense storms occurred during the deployment period with associated winds and rainfall creating a very robust and dynamic operational situation. With this in mind and in view of the data acquired from the remaining buoys the deployment was considered a success and proved the system’s viability overall.

During deployment 2, one buoy suffered slight water ingress due to poor glue application which affected some components after two weeks of operation. A second buoy ultimately failed after four weeks due to losing battery charge from persistent bird deposits on the solar panel. The mortality rate for each deployment was around 40% (i.e., 2 out of 5) in terms of the number of buoys that did not last the entire deployment duration. Ideally, for any size deployment over three months, we would aspire to reduce the mortality rate to <10% as the SEMAT system continues to evolve and becomes more robust.

In future deployments, we intend to increase the number of buoys to determine how the network performance scales with size. At present, the function of each buoy is independent of the others (i.e., no reliance on the others for transmission). It is only the web server that is the central point for potential congestion if too many devices are uploading data at the same time. Given the small number of devices and the asynchrony of the timing between buoys, this situation was not an issue for the aforementioned deployments.

7. *Transferability*—We proposed an open source standard for SEMAT to allow other individuals to expand upon this work and innovate around the design to ensure the project endures. We already have a newer model of SEMAT developed which will be documented in a future publication.

8. *Small environmental footprint*—The buoys used 40% recycled/upcycled e-waste components in the form of recycled laptop batteries and salvaged ABS plastic for 3D printer filament. After the buoys from the deployment were retrieved, many of the electronics components were salvageable and the 3D printed components could be recycled back into new 3D printer filament.

## 5. Conclusions

This paper presents the latest developments for the SEMAT project from a technological perspective and discusses a new community engagement paradigm for the development of environmental monitoring systems. We proposed the key goals for low-cost aquatic environmental monitoring, analysed the core hardware and software systems and presented a reproducible and simple design for SEMAT environmental monitoring buoys. Furthermore, the system has been developed using upcycled e-waste components and engages the community in its production through social enterprise such that its environmental footprint is greatly reduced and its gains extend beyond the technical and data domains to deliver social and economic benefits. 

The latest SEMAT buoys have been tested in a peri-urban lake setting to monitor water quality over several months as impacted by a nearby motorway, car park, golf course and residential properties. The field test demonstrates that the latest SEMAT buoys are well suited to this type of application and represent a validated niche technology that meets key stakeholder needs. This bodes well for the future of water quality monitoring across previously unaffordable geographic scales suited to a wider range of key stakeholders in the environmental management arena. Further, because of its low cost and potential for wider community uptake, workshops associated with the reported deployment have shown how effective the system is as an awareness and engagement tool for schools and the associated community networks. In this context, the SEMAT sensor network system has met all of the goals set for it to date.

Future work involves refining the electrical subsystems and physical design and the definition of a standard for a cleaning mechanism. Further field studies are planned as a combination of research, local government and private sector-based projects that will expand our operational experience and further refine systems design and performance.

## Figures and Tables

**Table 2:** Commercial-Based Water Quality Monitoring Products.

Vendor	Product(s)
AXYX Technologies [24]	TRIAXYS Directional Wave Buoy, TRIAXYS Wave and Current Buoy, TRIAXYS Mini Wave Buoy, HydroLevel Buoy
Fondriest Environmental [25]	NexSens Data Buoys
Aanderaa [26]	SeaGuard Platform
Fugro [27]	SEAWATCH Wavescan Buoy, SEAWATCH Midi Buoy, SEAWATCH Buoy, SEAWATCH Mini II Buoy
Aridea [28]	Libelium-Aridea Offshore Buoy Kit

**Table 3:** Electronics Subsystems.

Subsystem	Description
Microcontroller	System control
Power	Batteries and power management logic
Solar Charging	Solar panels and solar charge controller
Timing	Real time clock to control duty cycling
Sensors	Sensor head, connections, sampling, calibration
Communications	Transmissions of sensor samples and system status to the server
Cleaning	Self-cleaning mechanisms to reduce the impacts of bio fouling

**Table 4:** Price Comparison of SEMAT against Research and Commercial Buoys.

Manufacturer	Device	Cost
**Research Buoys**		
SEMAT	2018 Buoy	$154
Albaladejo et al. 2012 [30]	2011 Buoy	$395
Sadler et al. 2017 [9]	Telemeted Sensor System (not capable of deployment in water)	$181
Cave Pearl Logger [10]	Logging Device (no telemetry)	$100
GBROOS [14]		$50,000–$250,000
**Commercial Buoys**		
AXYS Technologies [24]	TRIAXYS Directional Wave Buoy, TRIAXYS Wave and Current Buoy, TRIAXYS Mini Wave Buoy, HydroLevel Buoy	Price only available upon request
Fondriest Environmental [25]	NetSense Data Buoys	$1495–$19,995
Aanderaa [26]	SeaGuard Platform	Price only available upon request
Fugro [27]	SEAWATCH Wavescan Buoy, SEAWATCH Midi Buoy, SEAWATCH Buoy, SEAWATCH Mini II Buoy	Price only available upon request
Aridea [28]	Libelium-Aridea Offshore Buoy Kit	$13,295
